# A Phase II Study of Sitravatinib Combined With Tislelizumab Plus Docetaxel for Acquired Resistance to PD‐(L)1 in Patients With Advanced/Metastatic Non‐Small Cell Lung Cancer

**DOI:** 10.1002/mco2.70538

**Published:** 2025-12-11

**Authors:** Yalun Li, Jin Zhou, Li Jiang, Hua Xie, Zonglian Gong, Ke Wang, Yan Zhang, Yan Li, Weimin Li, Panwen Tian

**Affiliations:** ^1^ Department of Pulmonary and Critical Care Medicine State Key Laboratory of Respiratory Health and Multimorbidity Precision Medicine Key Laboratory of Sichuan Province West China Hospital Sichuan University Chengdu China; ^2^ Lung Cancer Center/Lung Cancer Institute West China Hospital Sichuan University Chengdu China; ^3^ Department of Medical Oncology Sichuan Clinical Research Center for Cancer Sichuan Cancer Hospital & Institute Sichuan Cancer Center Affiliated Cancer Hospital of University of Electronic Science and Technology Chengdu China; ^4^ Department of Pulmonary and Critical Care Medicine Affiliated Hospital of North Sichuan Medical College Nanchong China

**Keywords:** acquired resistance, CD4^+^ T cell, immunotherapy, single‐cell secretome proteomics, TCR sequencing

## Abstract

In this Phase II study, 13 patients with stage IIIB/IV non‐small cell lung cancer with acquired resistance to immune checkpoint inhibitors were treated with tislelizumab, sitravatinib, and docetaxel. The efficacy and safety were evaluated. The combination treatment achieved median progression‐free survival of 7.6 months, median overall survival of 17.2 months, an objective response rate of 58.3% (1 not evaluable), and a disease control rate of 100%. Grade ≥ 3 treatment‐related adverse events were primarily neutropenia and leukopenia. Exploratory analyses showed a trend toward increased T cell receptor (TCR) diversity following treatment. High pre‐treatment CD8^+^ T cell polyfunctional strength index (PSI) showed a trend toward association with early treatment response and deeper tumor shrinkage, while CD8^+^ PSI decreased post‐therapy in responders, although not significantly. Larger changes in CD4^+^ T cell PSI were associated with longer PFS. Although these data indicate clinical benefit and immunologic correlates, the limited sample size precludes definitive conclusions. Furthermore, the observed changes in TCR and PSI dynamics are preliminary, hypothesis‐generating, and may provide insights for optimizing therapeutic strategies. However, validation via large‐scale, randomized controlled clinical trials remains warranted.

**Trial registration**: Chictr.org.cn, ChiCTR2200065547. Registered in 2022‐11‐08, https://www.chictr.org.cn/showproj.html?proj=183439.

## Graphical abstract

Over 60% of NSCLC patients treated with immune checkpoint inhibitors (ICIs) develop acquired resistance (AR). This study demonstrates that combining tislelizumab, sitravatinib, and docetaxel achieves an ORR of 58.3% and DCR of 100% in ICI‐resistant NSCLC. Median PFS was 7.6 months and median OS was 17.2 months. The change in CD4⁺ T cell polyfunctionality index before and after treatment may be potentially associated with patient survival.

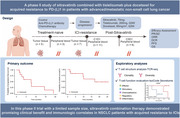

## 1 Introduction

Lung cancer remains the leading cause of cancer‐related mortality globally, with non‐small cell lung cancer (NSCLC) accounting for approximately 85% of all reported cases [1, 2]. The advent of immune checkpoint inhibitors (ICIs), particularly PD‐1/PD‐L1 blocking antibodies, has revolutionized the treatment of lung cancer by reactivating tumor‐infiltrating lymphocytes (TILs) and augmenting anti‐tumor immunity [3, 4]. However, drug resistance and an immunosuppressive tumor microenvironment have limited the use of ICIs [5, 6]. Notably, more than 60% of patients with NSCLC who initially respond to ICIs fail to sustain their response due to acquired resistance (AR), with some experiencing accelerated disease progression [7, 8, 9]. This necessitates the development of improved therapeutic strategies for NSCLC patients with AR.

Persistent tumor antigen stimulation and immune activation contribute to the development of an immunosuppressive microenvironment, which in turn promotes drug resistance [6, 10, 11]. Prior studies have elucidated potential mechanisms underlying AR, highlighting the interactions between T cells and tumor cells as major contributors [12, 13]. Sitravatinib, a receptor tyrosine kinase inhibitor (TKI), targets tumor‐associated macrophages (TAMs) through inhibition of TYRO3, AXL, MerTK, as well as receptors within the tyrosine kinase domain, including members of the vascular endothelial growth factor receptor (VEGFR) family. These targets are implicated in promoting an immunosuppressive tumor microenvironment [14]. When combined with PD‐1 blockade, sitravatinib has been shown to enhance cytotoxic T cell infiltration by converting TAMs from an immunosuppressive to an immunostimulatory phenotype [15].

A Phase Ib study demonstrated that the combination of sitravatinib and tislelizumab was generally well tolerated in patients with NSCLC, including those with anti–PD‐(L)1–resistant or refractory disease [16]. Objective responses were observed, with an overall response rate (ORR) of 8.7% in non‐squamous NSCLC and 18.3% in squamous NSCLC [16]. In another Phase Ib trial involving patients with advanced melanoma resistant or refractory to anti–PD‐(L)1 therapy, this combination also showed a manageable safety profile and promising antitumor activity [17].

This Phase II trial aimed to evaluate the efficacy and safety of sitravatinib combined with tislelizumab plus docetaxel in patients with advanced/metastatic NSCLC with AR to PD‐(L)1. Furthermore, patient samples were collected longitudinally to perform T cell receptor (TCR) sequencing and single‐cell secretome proteomics to evaluate structural and functional alterations in TCRs and the secretory abilities of CD4^+^ and CD8^+^ T cells. Our data preliminarily observe a trend toward increased TCR diversity, a possible association between pre‐treatment CD8^+^ T cell polyfunctional strength index (PSI) and depth of response, and between changes in CD4^+^ T cells and improved progression‐free survival (PFS). These exploratory findings imply that T cell dynamics and the distinct roles of CD4^+^ and CD8^+^ T cells may contribute to the immune response landscape during post‐AR treatment, providing preliminary insights for future investigations.

## 2 Results

### 2.1 Sitravatinib Combination Therapy Mitigated ICI Resistance in Patients With NSCLC

From February 1, 2023, to November 3, 2023, 13 patients were enrolled in the study, with a median age of 56 years (range 32–70), 84.6% male, and 69.2% presenting with stage IV disease. Histologically, 46.2% of the patients had squamous cell carcinoma, and 53.8% had non‐squamous cell carcinoma. All patients had previously received concomitant anti‐PD‐(L)1 therapy combined with platinum‐based chemotherapy before initiating the study treatment. Of these, 46.2% had received ICI treatment for ≥ 6 months prior to disease progression (PD), while 53.8% had received ICI treatment for < 6 months prior to PD. The baseline characteristics of the study participants are summarized in Table 1.

**TABLE 1:** The characteristics of the patients enrolled in the trial.

	*N* (%) *n* = 13
**Age, median (range)**	56 (32–70)
**Sex**	
Male	11 (84·6)
Female	2 (15·4)
**Histology**	
Nonsquamous	7 (53·8)
Squamous	6 (46·2)
**ECOG PS**	
0	2 (15·4)
1	11 (84·6)
**Smoke**	
Current or former	11 (84·6)
Never	2 (15·4)
**Stage**	
IIIB	4 (30·8)
IV	9 (69·2)
**Site of metastases**	
Brain	2 (15·4)
Liver	2 (15·4)
Bone	2 (15·4)
**Prior NSCLC treatment**	
Tislelizumab + platinum‐based doublet chemotherapy	6 (46.1)
Sintilimab + platinum‐based doublet chemotherapy	4 (30.8)
Pembrolizumab + platinum‐based doublet chemotherapy	1 (7.7)
Atezolizumab + platinum‐based doublet chemotherapy	1 (7.7)
Camrelizumab + platinum‐based doublet chemotherapy	1 (7.7)
**Duration of prior ICI treatment to PD**	
≥6 months	6 (46·2)
<6 months	7 (53·8)

Abbreviations: ECOG PS, Eastern Cooperative Oncology Group performance status; NSCLC, non‐small cell lung cancer; ICI, immune checkpoint inhibitor; PD, progression disease.

**FIGURE 1:**
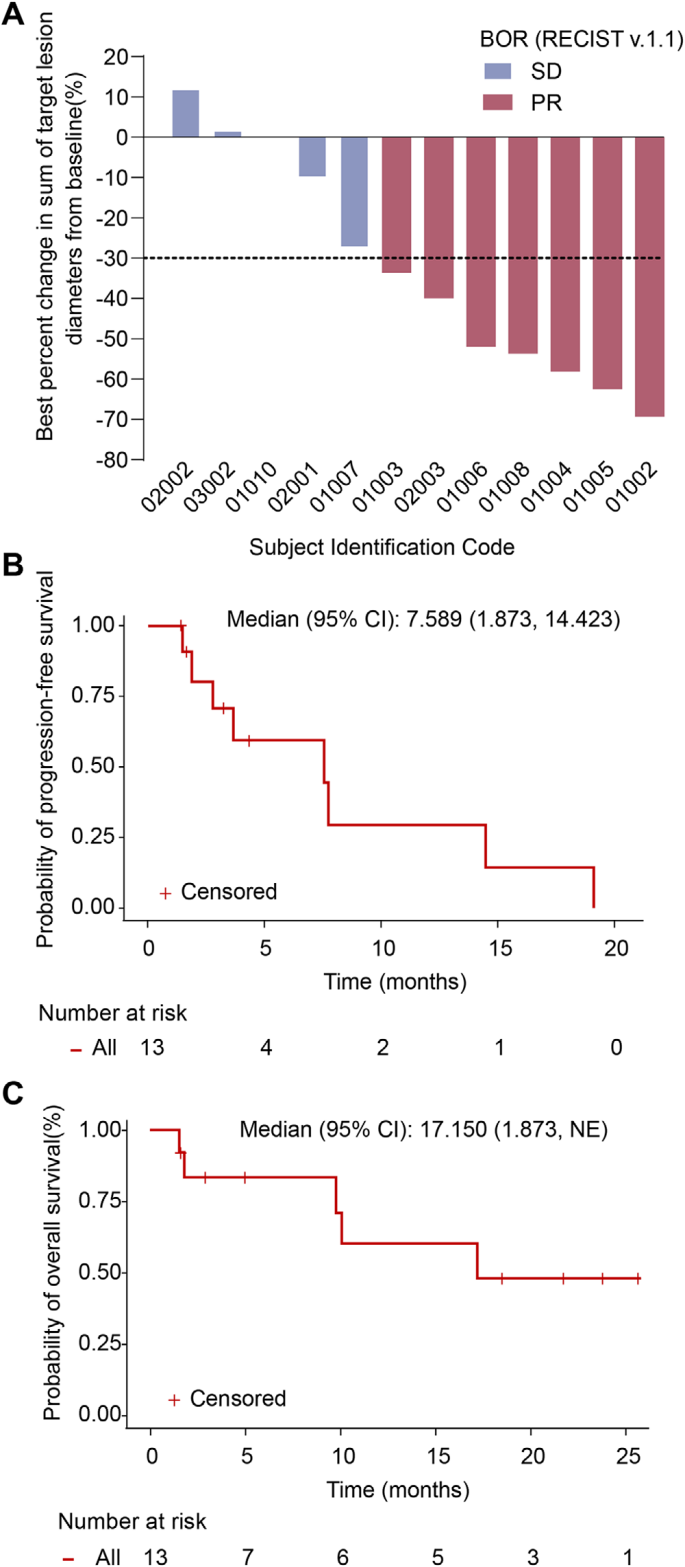
Tumor response and survival outcomes. (A) Best percent change in sum of target lesion diameters from baseline. Data cutoff: November 3, 2023. (B) Progression‐free survival and (C) overall survival in patients with advanced NSCLC who progressed on prior PD‐1 inhibition and chemotherapy (*n* = 13).

Among the 13 patients, 12 were evaluable for efficacy, among whom 7 achieved a partial response (PR) as their best response, and the remaining patients achieved stable disease (SD). The ORR was 58.3%, and the disease control rate (DCR) was 100% (Table 2, Figure 1A). Median PFS was 7.6 months (95% confidence interval [CI] 1.9 to 14.4, Figure 1B). The estimated proportions of patients alive and progression‐free at 6 and 12 months were 58.9% (95% CI 23.4% to 82.5%) and 29.5% (95% CI 4.8% to 61.2%), respectively. Median overall survival (OS) was 17.2 months (95% CI 1.9, not reached) (Figure 1C). The estimated proportion of patients who were alive at 12 months was 59.9% (95% CI 23.8% to 83.3%).

**TABLE 2:** Summary of the evaluation of treatment response.

	*N* = 12
**BOR, *n* (%)**	
PR	7 (58·3%)
SD	5 (41·7%)
**ORR, *n* (%)**	7 (58·3%)
**DCR, *n* (%)**	12 (100%)

Abbreviations: BOR, best overall response; PR, partial response; SD, stable disease; ORR, objective response rate; DCR, disease control rate.

Regarding safety, the incidence of treatment‐related adverse events (TRAEs) was 100% (13/13), with grade 3–5 TRAEs in 92.3% (12/13) of patients. Most grade ≥ 3 adverse events were neutropenia and leukopenia. The incidence of serious adverse events (SAEs) was 46.2%, and 30.8% of the patients experienced immune‐related adverse events. The detailed safety profile of the study treatment is shown in Table S1.

### 2.2 Dynamic Changes in the TCR Repertoire of ICI‐Resistant NSCLC Before and After Sitravatinib Treatment

**FIGURE 2:**
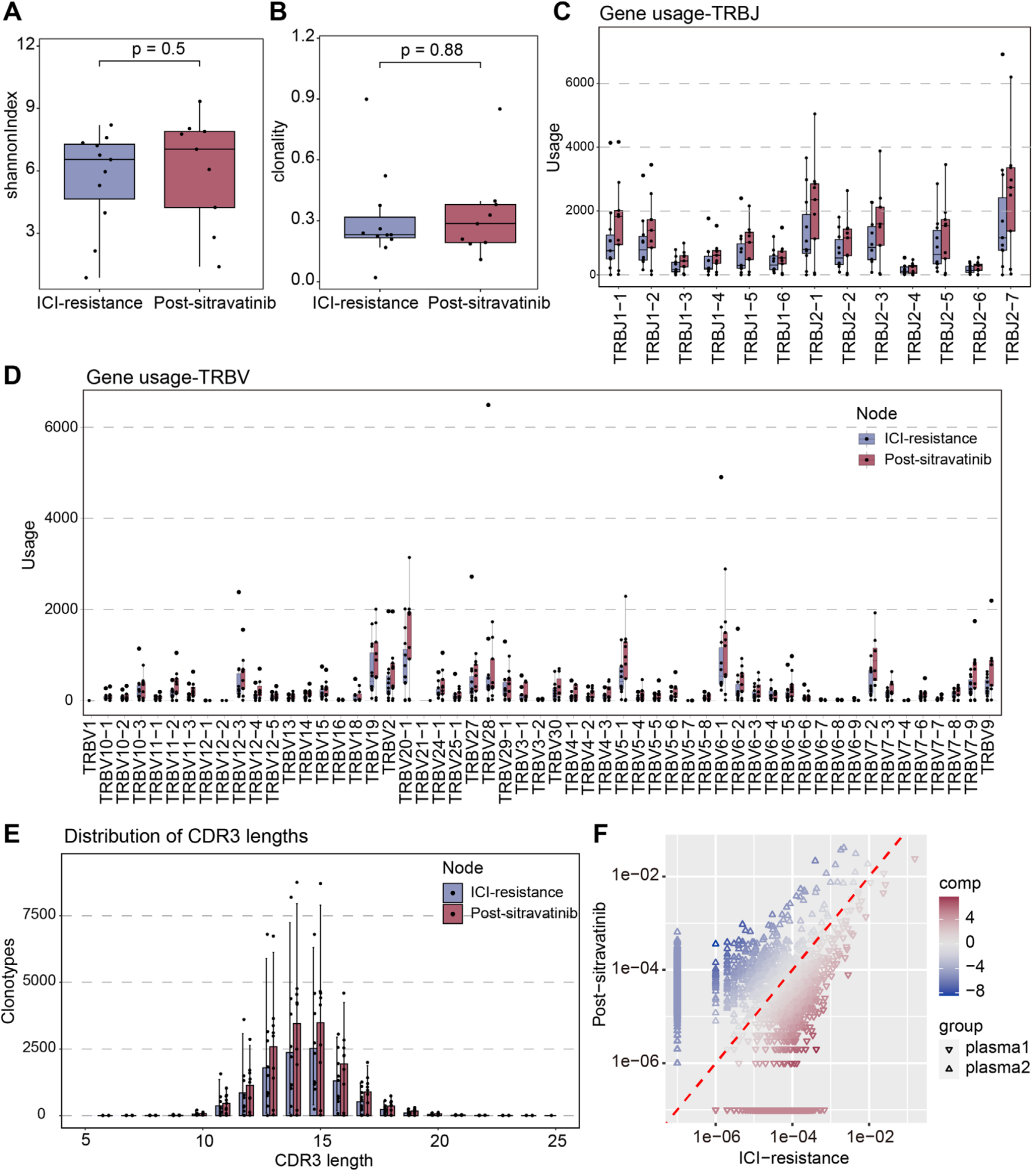
Analysis of T cell repertoire before and after sitravatinib treatment. Comparison of (A) clonality index and (B) Shannon diversity index in plasma samples from NSCLC patients with ICI resistance before (*n* = 11) and after (*n* = 9) sitravatinib treatment. (C) Bar charts showing the comparison of TRBJ and (D) TRBV gene usage frequencies between the ICI‐resistant (*n* = 11) and the post‐sitravatinib (*n* = 9) groups from plasma samples. (E) Bar chart displaying the distribution of CDR3aa sequence lengths between the ICI‐resistant (*n* = 11) and post‐sitravatinib (*n* = 9) groups from plasma samples. (F) Scatter plot for a representative patient (Patient 01002), illustrating the distribution of clones between paired plasma samples (*n* = 6) from NSCLC patients with ICI resistance before and after sitravatinib treatment.

We conducted comprehensive TCR sequencing to analyze the TCR gene repertoire and investigate the clonal response of anti‐tumor T cells. Compared with the treatment‐naïve group (before immunotherapy), Shannon diversity was significantly reduced (median difference ‐1.227, 95% CI ‐3.155 to ‐0.175, *p* = 0.048) in the ICI‐resistant group (Figure S1A). Following sitravatinib treatment, a median change in Shannon diversity was +0.545 (*p* = 0.5, Figure 2A). No changes in clonality were observed (Figures S1B and 2B).

TCRs are composed of α and β chains, with diversity generated through the rearrangements of V (variable), D (diverse), and J (joining) gene segments, as well as random nucleotide insertions or deletions. We compared the usage patterns of T cell receptor β variable (TRBV) and joining (TRBJ) gene segments within the TCR repertoire.

In the ICI‐resistant group, the frequencies of TRBJ and TRBV usage were lower than in the treatment‐naïve group (Figure S1C, D). Following treatment with sitravatinib, the frequencies of TRBJ and TRBV usage increased (Figure 2C, D). The most frequently observed TRBJ/TRBV segments, such as TRBJ2‐7, TRBJ2‐1, TRBV6‐1, and TRBV20‐1, were consistent with findings in paired PSCC tumors and adjacent normal tissues [18].

The CDR3 region plays a direct role in antigen recognition. Compared to the treatment‐naïve group, the CDR3 sequences in the ICI‐resistant group were shorter (Figure S1E). In contrast, the post‐sitravatinib treatment group showed an increase in CDR3 sequence length (Figure 2E). Although most CDR3 amino acid (CDR3aa) sequences were unique to individual patients (Figure S2A), a subset of shared motifs was identified across patients within both the ICI‐resistant (≥ 5/11 patients) and post‐sitravatinib (≥ 5/9 patients) groups. This suggests limited but detectable clonal overlap among patients (Figure S2B). Among them, the CASSLGETQYF sequence exhibited a trend toward downregulation in the post‐sitravatinib group (Figure S2C). We further investigated the frequency changes in T cell populations with similar TCR specificities in paired samples from six patients. A substantial proportion of shared CDR3aa clonotypes was observed between pre‐ and post‐sitravatinib treatment samples from the same patient (Figures 2F and S2D), indicating persistence of antigen‐experienced T cell clones.

Overall, sitravatinib treatment in patients with NSCLC appears to remodel the T cell repertoire by influencing TCR diversity, including changes in TRBV and TRBJ gene usage patterns and CDR3 sequence characteristics.

### 2.3 Differential PSI Response of CD4^+^ and CD8^+^ T Cells in ICI‐Resistant NSCLC Before and After Sitravatinib Treatment

We analyzed the secretion of 32 cytokines by CD4^+^ and CD8^+^ T cells using single‐cell analysis to evaluate their polyfunctionality (defined as the secretion of ≥2 cytokines) in peripheral blood samples from patients.

No significant differences were observed between the ICI‐resistant and treatment‐naïve groups in both PSI and polyfunctionality of CD4^+^ (median PSI 121.3 vs. 126.4; median polyfunctionality, 6.94 vs. 6.385, *p* = 0.5476) and CD8^+^ T cells (median PSI, 88.21 vs. 357.9; median polyfunctionality, 4.61 vs. 16.13, *p* = 0.5476) (Figure S3A‐F).

**FIGURE 3:**
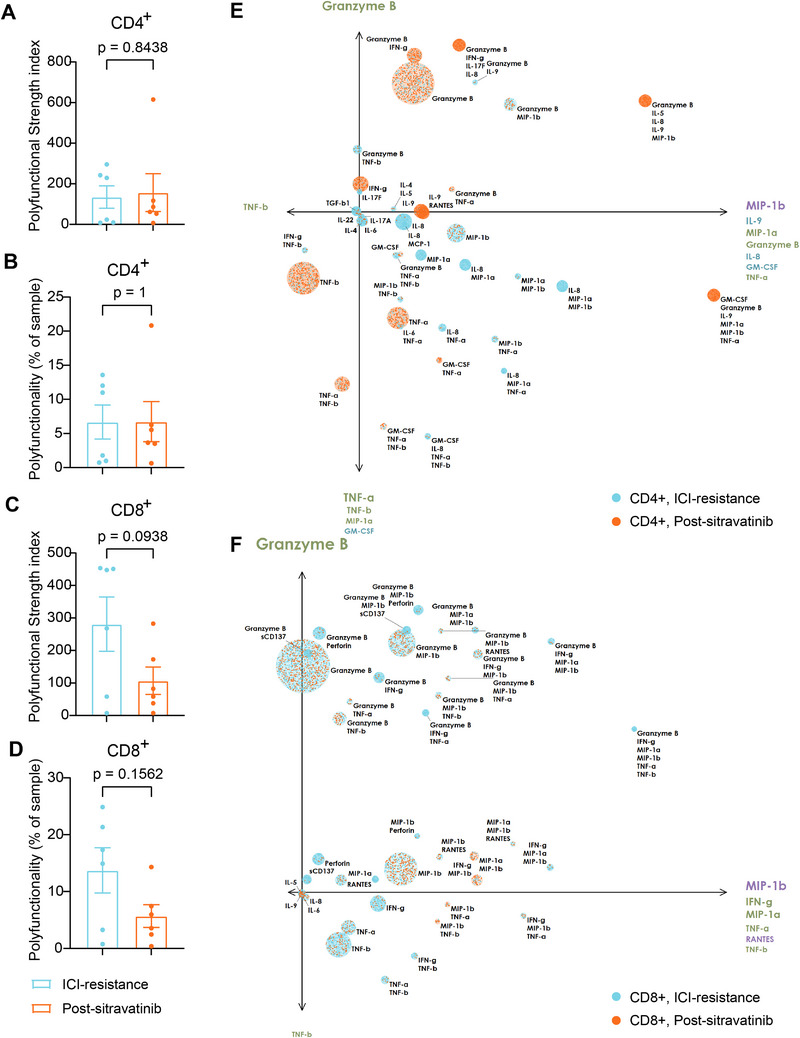
Polyfunctionality analysis of CD4^+^ and CD8^+^ T cells before and after sitravatinib treatment. (A) Comparison of the polyfunctional strength index (PSI) of peripheral CD4^+^ T cells in the ICI‐resistant and post‐sitravatinib groups. (B) Comparison of the polyfunctionality of peripheral CD4^+^ T cells. (C) Comparison of the PSI of peripheral CD8^+^ T cells in the ICI‐resistant and post‐sitravatinib groups. (D) Comparison of the polyfunctionality of peripheral CD8^+^ T cells. PAT‐PCA plots illustrating the distribution of polyfunctional (E) CD4^+^ and (F) CD8^+^ T cell subsets and their dynamic shifts. Larger circles represent a higher number of polyfunctional subpopulations secreting specific cytokines. Each point represents a single cell, while circles denote subpopulations with similar secretion profiles. The overall color of each group corresponds to the color of the sample that secreted the group with the highest frequency. The factors labelled along the axis arrows indicate the main drivers of differences between the samples. *N* = 6.

Following sitravatinib treatment, CD4⁺ and CD8⁺ T cells exhibited distinct trends in functional changes. Specifically, CD4^+^ T cell PSI (median difference 40.79; *p* = 0.84) and polyfunctionality (median difference 2.330; *p* = 1) showed no significant changes post‐treatment, although a numerical upward trend was observed in 66.6% patients (4/6) (Figures 3A, B and S4A, B). This subtle change was primarily associated with alterations in the polyfunctional subsets (secreting ≥ 4 cytokines) (Figure S4B). The median PSI of regulatory CD4^+^ T cells decreased from 1.265 to 0.225 post‐treatment (95% CI ‐22.08 to 0.45, *p* = 0.1250), suggesting a trend toward alleviation of immunosuppression. The PSI (median difference ‐190.2; 95% CI ‐443.5 to 74.83; *p* = 0.0938) and polyfunctionality (median difference ‐7.185; 95% CI, ‐24.46 to 5.19; *p* = 0.1562) of CD8^+^ T cells also showed a decreasing trend post‐therapy (Figures 3C, D and S4C, D). PCA of polyfunctional CD4^+^ and CD8^+^ subsets revealed similar patterns (Figure 3E, F). These results highlight heterogeneity in the polyfunctionality of single CD4^+^ and CD8^+^ T cells, as well as distinct roles for these cell populations in mitigating ICI resistance.

### 2.4 Association of Polyfunctional CD4^+^ and CD8^+^ T Cells With Response to Sitravatinib Treatment

**FIGURE 4:**
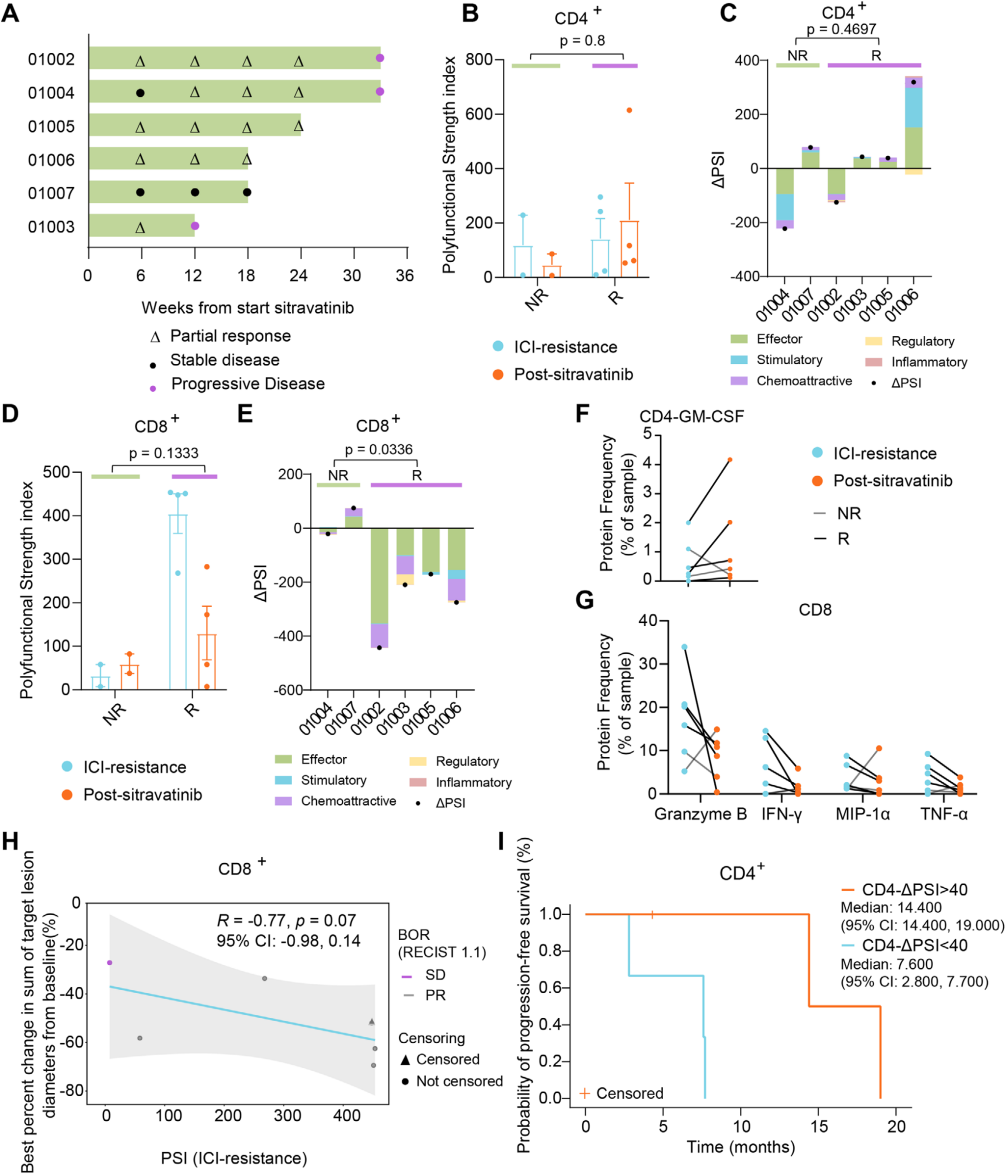
Analysis of T cell polyfunctionality and efficacy. (A) Swimmer plot showing the treatment duration and clinical responses of individual patients. Patients were stratified into non‐responders (NR, SD, *n* = 2) and responders (R, PR, *n* = 4) based on their clinical response at C3D1 (6 weeks after sitravatinib treatment). (B) Comparison of the polyfunctional strength index (PSI) of peripheral CD4^+^ T cells in non‐responders and responders. Each dot indicates an individual patient. (C) Comparison of changes in PSI (ΔPSI) of CD4^+^ T cells between non‐responders and responders. Each bar chart indicates an individual patient. (D) Comparison of the PSI of peripheral CD8^+^ T cells. (E) Comparison of ΔPSI of CD8^+^ T cells. (F) The protein frequency of GM‐CSF production by CD4^+^ T cells. Blue dots indicate ICI‐resistant, orange dots indicate post‐sitravatinib, gray lines represent non‐responders, and black lines represent responders. (G) The protein frequency of granzyme B, IFN‐γ, MIP‐1α, and TNF‐α production by CD8^+^ T cells. (H) Spearman correlation between pre‐treatment PSI of CD8⁺ T cells and the depth of tumor response to sitravatinib. (I) Kaplan–Meier analysis of progression‐free survival stratified by changes in CD4⁺ T cell PSI (ΔPSI) following sitravatinib combination therapy.

Patients were stratified into two groups based on their clinical response on the first day of the third treatment cycle (C3D1, 6 weeks after sitravatinib treatment): non‐responders (SD, *n* = 2) and responders (PR, *n* = 4) (Figure 4A). This grouping was used to evaluate differences in dynamic changes of PSI (ΔPSI) and polyfunctionality (ΔPoly) before and after treatment. The ΔPSI values of CD4^+^ T cells in the two non‐responders were –222.4 and 78.18, respectively, whereas those in the responders were 40.79 (median; range ‐125.2 to 319.5). No systematic differences were observed between the two groups (Figure 4B, C).

In CD8^+^ T cells, responders exhibited numerically higher PSI values at the ICI‐resistant stage (median 449.4; range 268.0–453.5) than non‐responders (7.49 and 58.22) (Figure 4D). The ΔPSI values for non‐responders were ‐20.59 and 74.83, whereas responders showed a consistent numerical decrease (median difference: ‐242.5; range: ‐443.5 to ‐170.6) (Figure 4E). CD8⁺ T cell PSI declined more substantially in responders (Mann–Whitney test, *p* = 0.1333; mixed‐effects model, *p* = 0.0336). Further analysis of cytokine secretion frequencies revealed that, in responders, post‐treatment CD4^+^ T cells displayed reduced GM‐CSF secretion, while CD8⁺ T cells exhibited decreased secretion of granzyme B, IFN‐γ, MIP‐1α, and TNF‐α, showing a concordant directional pattern across all four responders (Figure 4F, G). This consistent immunological pattern in responders suggests a link between sitravatinib's immunomodulatory effects and its clinical efficacy.

The correlation between CD8^+^ PSI before sitravatinib treatment and depth of response was assessed using Spearman correlation analysis (*R* = ‐0.77, *p* = 0.07) (Figure 4H). Furthermore, patients with a greater change in CD4^+^ PSI following sitravatinib treatment (ΔPSI >40) exhibited a longer median PFS (14.4 vs. 7.6 months; log‐rank test, *p* = 0.0499, Figure 4I). These preliminary findings suggest that CD8⁺ T cell PSI may be associated with therapeutic benefit in overcoming ICI resistance, with higher pre‐treatment CD8⁺ PSI correlating with better tumor response. In contrast, changes in CD4⁺ PSI (ΔPSI) following treatment may indicate improved survival outcomes. Given the limited sample size, these observations warrant further validation in larger cohorts.

## 3 Discussion

In this study, we determined whether the sitravatinib‐related combination regimen could help overcome AR to ICIs in patients with advanced or metastatic NSCLC. To our knowledge, this is the first report of a multi‐targeted TKI combined with a PD‐1 inhibitor plus chemotherapeutic agent in ICI‐resistant advanced/metastatic NSCLC. Encouraging anti‐tumor activity was observed in this preliminary and limited cohort study.

Patients with ICI‐resistant advanced NSCLC exhibit poor prognosis, with a median OS of 10–12 months. Clinical evidence supporting the reintroduction of ICIs in NSCLC patients following progression on prior PD‐(L)1 therapy remains limited. Combination approaches involving multi‐targeted TKIs and PD‐1 inhibitors have demonstrated modest efficacy. In the Phase II MRTX‐500 study, sitravatinib (120 mg daily) plus PD‐1 inhibitor nivolumab achieved an ORR of 16.9% in ICI‐experienced patients with NSCLC who previously benefited clinically, with median PFS and OS of 5.6 and 13.6 months, respectively [19]. Similarly, in another Phase II study, sitravatinib (120 mg daily) combined with tislelizumab resulted in ORRs of 8.7% and 18.2% in patients with anti‐PD‐(L)1 resistant/refractory nonsquamous and squamous NSCLC (PFS, 4.2 and 5.3 months; OS, 10.1 and 10.5 months) [16], respectively. Docetaxel remains a standard treatment option for patients with advanced NSCLC who have previously received both ICI therapy and platinum‐based doublet chemotherapy [20], although its efficacy remains insufficient. In the Phase III SAPPHIRE study, sitravatinib combined with the PD‐1 inhibitor nivolumab numerically prolonged OS compared with docetaxel monotherapy (12.2 vs. 10.6 months) [21]. Rationally designed combination strategies may provide more effective treatment options.

Previous research evaluating patients with prior ICI exposure and disease progression in Phase II and III randomized trials has included several treatment strategies [22]. Among these, TKI+chemotherapy demonstrated the best outcomes in PFS and ORR, while ICI+TKI showed the most favorable OS, highlighting the potential of TKI combination therapies. Herein, the triplet regimen of sitravatinib, tislelizumab, and docetaxel yielded a promising ORR of 58.3% and a median PFS of 7.6 months. Although high rates of TKI‐related adverse events may hinder further development, balancing dose, efficacy, and safety remains a key consideration. Overall, although sitravatinib is no longer in clinical development, our exploratory clinical data suggest that a multitargeted TKI combined with PD‐1 blockade and chemotherapy may offer a new therapeutic option for patients with NSCLC who have developed AR to ICIs. Further investigation of similar strategies in larger prospective trials remains warranted.

In this study, the combination therapy showed no new safety signals; however, it demonstrated limited tolerability. Grade ≥ 3 TRAEs were primarily hematologic, including neutropenia and leukopenia, which are attributable to docetaxel toxicity and may be associated with increased susceptibility in Asian populations [23]. The incidence of other grade ≥ 3 TRAEs, such as hypertension and pneumonia, was consistent with that reported in the SAPPHIRE study [21] and the SAFFRON‐301 study [24].

TCR diversity and T cell cytokine production are critical markers for immune responses against tumors and pathogens [25]. Persistent antigen stimulation may lead to overexpansion of certain T cell clones, depleting or exhausting other clones, and selectively reducing TCR diversity [26]. This phenomenon might play a role in ICI resistance, as patients with greater homology in tumor‐adjacent tissue TCRs often show weaker antitumor immune responses and have lower survival rates [27]. We observed lower TCR diversity in ICI‐resistant NSCLC, which may be linked to the immunosuppressive tumor microenvironment [28]. Following sitravatinib combination therapy, increased TCR diversity in patients with mitotic drug resistance was supported by enhanced TRBV/TRBJ usage and longer CDR3 sequences. Sitravatinib, by modulating the tumor immune microenvironment and reducing immunosuppressive cell populations [29, 30], may help restore T cell repertoire diversity and potentially reactivate new antigen‐specific T cells.

Polyfunctionality, a key indicator of T cell efficacy, is closely associated with immune regulation in response to viruses and tumors [31]. Previous studies have demonstrated distinct roles for CD8^+^ and CD4^+^ T cells in the ICI response in NSCLC. CD8^+^ T cells primarily activate anti‐tumor immunity, whereas CD4^+^ T cells are more likely to contribute to sustained immune responses and long‐term survival [32, 33, 34, 35]. Consistent with these findings, our research revealed that the polyfunctionality of CD4^+^ and CD8^+^ T cells showed distinct trends in patients with NSCLC who achieved remission of ICI resistance. To further explore the clinical significance of these changes, we performed a preliminary stratification based on treatment response.

Responders exhibited higher pre‐treatment CD8^+^ T cell PSI values (median: 449.4) than non‐responders (7.49 and 58.22), suggesting that these CD8^+^ T cells were more likely to be functionally suppressed than exhausted or senescent [36]. The potential relationship between pre‐treatment PSI and response depth was observed, although not significant (*R* = ‐0.77, *p* = 0.07). The PSI of CD8^+^ T cells in ICI‐resistant NSCLC may help identify patients more likely to benefit from treatment. We observed a more pronounced decline in PSI of CD8^+^ T cells in responders following sitravatinib treatment, which contrasts with previous observations in ICI‐naïve patients with NSCLC [33]. This decline may reflect active anti‐tumor effector responses by CD8⁺ T cells, as evidenced by changes in effector cytokines, such as Granzyme B and IFN‐γ. In ICI‐resistant patients, such functional shifts in T cells are often linked to tumor specificity, chronic antigen re‐exposure, epigenetic remodeling, and metabolic state [36]. The specific mechanisms underlying this change warrant further investigation. Our study also revealed that patients with higher ΔPSI tended to have longer PFS. In NSCLC, dynamic changes in CD4⁺ T cell subsets have been observed between ICI‐resistant and post‐resistance remission states [37, 38]. CD4^+^ T cells may be critical for overcoming ICI resistance [39, 40], and their ΔPSI is a potential biomarker for immunotherapeutic outcomes in NSCLC [33]. These observations are exploratory and hypothesis‐generating, and whether similar PSI dynamics occur under other treatment settings remains to be determined. Further validation in larger, prospective studies is warranted.

### 3.1 Limitations

Our study has some limitations. First, this Phase II study was a single‐arm trial without a randomized control group, which may introduce potential bias from historical comparisons and reduce the strength of evidence. Second, the study was terminated early due to the discontinuation of sitravatinib development. Furthermore, the relatively small sample size might have introduced selection bias affecting the estimation of efficacy and limited the feasibility of further subgroup analyses. Moreover, the small sample size also reduced the statistical power; however, we have endeavored to present the data as objectively as possible to minimize bias. Third, IsoCode analysis, initiated with in vitro secretome activity, may not fully recapitulate the complexity of T cell responses within the native tumor microenvironment [41]. Fourth, we did not distinguish between the antigen specificity of CD4^+^ and CD8^+^ T cells, which exhibit inherent functional and subset differences [42].

## 4 Conclusion

This Phase II study evaluated the efficacy and safety of sitravatinib combined with tislelizumab and docetaxel in patients with advanced or metastatic NSCLC who had AR to ICIs, providing preliminary insights into the exploration of similar combination strategies. Although the ORR (58.3%) and median PFS (7.6 months) appeared numerically promising, the limited sample size precludes definitive conclusions. The observed changes in TCR diversity and the polyfunctionality of CD4^+^ and CD8^+^ T cells following combination therapy suggest a potential immunomodulatory mechanism underlying the reversal of AR to ICIs in NSCLC. These immune features warrant further validation in large‐scale, randomized controlled clinical trials. This will enable more patients to benefit from such therapeutic approaches.

## 5 Materials and Methods

### 5.1 Study Design and Participant Details

This single‐arm, multicenter Phase II trial, registered at Chictr.org.cn (ChiCTR2200065547), evaluated the efficacy and safety of a PD‐1 inhibitor plus sitravatinib, combined with a limited course (2 cycles) of docetaxel, in patients with advanced NSCLC who progressed on prior PD‐1 inhibition and chemotherapy. Eligible patients were aged 18–75 years with a histologically or cytologically confirmed diagnosis of locally advanced or metastatic NSCLC without targetable driven mutations. Participants were required to have progressed on either a combination of anti‐PD‐(L)1 antibody and platinum‐based chemotherapy or on platinum‐based chemotherapy following the failure of anti‐PD‐(L)1 antibody monotherapy. Additional inclusion criteria included an Eastern Cooperative Oncology Group (ECOG) performance status (PS) of 0 or 1 and an adequate organ function. Patients with prior docetaxel therapy, anti‐angiogenic drug therapy, prior immunotherapy other than anti‐PD‐(L)1 antibody therapy, or unacceptable toxicity following prior anti‐PD‐(L)1 antibody therapy were excluded from the study. Participants received tislelizumab 200 mg intravenously every 3 weeks in combination with sitravatinib 70 mg orally once daily and docetaxel 60 mg/m^2^ intravenously every 3 weeks (2 cycles) until disease progression, intolerable toxicity, death, withdrawal of informed consent, whichever occurs first.

### 5.2 End Points

The primary endpoint is a 6‐month PFS rate. Secondary endpoints include PFS, ORR, DCR, and OS. Tumor imaging was assessed according to the RECIST v.1.1 criteria. Tumor assessments were scheduled every 6 weeks for the first 25 weeks and then every 9 weeks during the study period, regardless of whether a treatment dose delay occurred. The incidence and severity of adverse events were a secondary safety endpoint. Safety and tolerability were assessed throughout the trial by monitoring AEs, defined and graded according to the National Cancer Institute Common Terminology Criteria for Adverse Events version 5.0.

The sample size was determined based on the primary end point (6‐month PFS rate) using a historical control of 26% in the chemotherapy arm from the RATIONALE‐303 study. The study aimed to enroll 43 patients but was terminated early due to the discontinuation of sitravatinib development. The number and proportion of patients with rate endpoints (ORR and DCR) were summarized. The Kaplan–Meier method was used to estimate the curves for the time‐to‐event variables (PFS and OS) and their corresponding quartiles (including the median).

### 5.3 TCR Sequencing

Peripheral blood samples were collected to perform TCR sequencing at two time points: prior to the initiation of treatment (cycle 1, day 1 [C1D1], ICI‐resistant group, *n* = 11) and on C3D1 (post‐sitravatinib group, *n* = 6). Additionally, tumor tissue samples (formalin‐fixed, paraffin‐embedded, FFPE) were collected from patients in two reference groups: the treatment‐naïve group (before immunotherapy, *n* = 5) and the ICI‐resistant group (*n* = 7). DNAs were extracted using the DNA FFPE Kit and the QIAamp DNA Blood Kit (Qiagen). Multiplex primers were employed to amplify the CDR3 regions of the V and J gene segments from genomic DNA. Paired‐end 100 bp PCR products were generated and sequenced on an Illumina platform. TCR‐Vβ sequencing was conducted in a CLIA/CAP‐certified laboratory (Geneplus), with low‐quality sequences filtered out during post‐sequencing quality control. The V and J gene segments were aligned using MIXCR [43] and annotated with the IMGT (ImMunoGeneTics) database to identify TCR CDR3 sequences. TCR data analysis was performed using the immunarch package, and visualizations were generated using R.

### 5.4 IsoCode Single‐Cell Secretome Proteomics

Single‐cell secretome proteomics, an advanced analytical technique that enables multiparametric protein characterization of viable, protein‐secreting individual cells, was employed to assess T cell function. Peripheral blood samples from the treatment‐naïve group (*n* = 3), the ICI‐resistant group (*n* = 6), and the post‐sitravatinib group (*n* = 6) were used to assess T cell function. Cryopreserved patient peripheral blood mononuclear cell samples were thawed and cultured in complete RPMI medium (GIBCO) supplemented with IL‐2 (10 ng/mL; Biolegend) at a density of 1 × 10^6^/mL in a 37°C, 5% CO_2_ incubator overnight. Following recovery, CD4^+^/CD8^+^ T cell subsets were enriched using anti‐CD4 or anti‐CD8 microbeads (Miltenyi Biotec). The cells were then stimulated with immobilized anti‐CD3 antibody (10 µg/mL; CD3 Monoclonal Antibody (OKT3), ThermoFisher Scientific) and anti‐CD28 antibody (5 µg/mL; CD28 Monoclonal Antibody (CD28.2), ThermoFisher Scientific) at 37°C, 5% CO_2_ for 24 h. Subsequently, the cells were stained with Cell Stain 405 dye (IsoPlexis). Approximately 30,000 cells per sample were loaded on the IsoCode single‐cell chip (IsoPlexis). For each sample, protein secretions from approximately 1000 single cells were captured using a 32‐plex antibody barcoded chip and analyzed [44].

The polyfunctionality of T cells, that is, the ability of a single cell to secrete ≥ 2 cytokines, was assessed using IsoSpeak software (IsoPlexis). The PSI of T cells was calculated as the product of the percentage of polyfunctional cells and the mean fluorescence intensity of the proteins secreted by these cells [45, 46]. Polyfunctional heatmaps were generated to illustrate the heterogeneity and diversity in cytokine secretion profiles across different cell samples. To further analyze the data, dimensionality reduction was performed, and PAT‐PCA plots were utilized to visually depict variations in secretion patterns among the samples.

### 5.5 Statistical Analyses

The generalized Brookmeyer and Crowley method [47] was used to estimate two‐sided 95% CIs for the median values. One‐year PFS and OS rates, along with the corresponding 95% CIs, were estimated using the Greenwood formula [48]. The ORR and DCR were calculated using the Clopper–Pearson method. Two‐sided 95% CIs for ORR and DCR were computed using the Pearson method to assess the precision of the point estimates. Given the limited sample size, group comparisons in TCR sequencing and IsoCode single‐cell secretome proteomics analyses were performed using the Wilcoxon rank‐sum test. Data are presented as mean ± standard error.

## Author Contributions

Conception and design: Yalun Li, Weimin Li, Panwen Tian. Administrative support: Weimin Li. Provision of study materials or patients: Jin Zhou, Hua Xie, Li Jiang, Zonglian Gong, Ke Wang, Yan Zhang; Yan Li. Collection and assembly of data: Yalun Li, Panwen Tian. Data analysis and interpretation: Yalun Li, Weimin Li, Panwen Tian. Manuscript writing: all authors. All authors have read and approved the final manuscript.

## Funding

This work was supported by the National Natural Science Foundation of China (No. 82473213 to P Tian, 82470099 to Y Li, 92159302 to W Li).

## Ethics Statement

This study was approved by the Biomedical Research Ethics Committee of West China Hospital of Sichuan University [No. 2022‐(1369)]. Written informed contents were obtained from every participant. The study was registered at Chictr.org.cn (ChiCTR2200065547).

## Conflicts of Interest

The authors have declared that no conflict of interest exists.

## Supporting information




**Figure S1**: Analysis of T cell repertoire before and after immunotherapy. (A) Comparison of clonality index and (B) Shannon diversity index in tissue samples from NSCLC patients in the treatment‐naïve (*n* = 5) and ICI‐resistant (*n* = 7) groups. (C) Bar charts showing the comparison of TRBJ and (D) TRBV gene usage frequencies between the treatment‐naïve (*n* = 5) and ICI‐resistant (*n* = 7) groups from plasma samples. (E) Bar chart displaying the distribution of CDR3aa sequence lengths between the treatment‐naïve (*n* = 5) and ICI‐resistant (*n* = 7) groups from tissue samples.
**Figure S2**: Analysis of CDR3 amino acid sequences. (A) Bar chart showing the number of shared CDR3aa sequences derived from varying numbers of samples across different patients in the ICI‐resistant and post‐sitravatinib groups. (B) Representative motifs of shared CDR3aa sequences in the ICI‐resistant and post‐sitravatinib groups. (C) Bar chart comparing the frequencies of major shared CDR3aa motifs in paired patient samples between the ICI‐resistant and post‐Sitravatinib groups. (D) Scatter plots illustrating the distribution of clones between the ICI‐resistant and post‐Sitravatinib groups from plasma samples, presented sequentially for patients 01003, 01004, 01005, 01006, and 01007.
**Figure S3**: Polyfunctionality analysis of CD4^+^ and CD8^+^ T cells before and after immunotherapy treatment. (A) Comparison of the polyfunctional strength index (PSI) of peripheral CD4^+^ T cells in the treatment‐naïve and ICI‐resistant groups. (B) Comparison of the polyfunctionality of peripheral CD4^+^ T cells. (C) Comparison of the PSI of peripheral CD8^+^ T cells in the treatment‐naïve and ICI‐resistant groups. (D) Comparison of the polyfunctionality of peripheral CD8^+^ T cells. PAT‐PCA plots illustrating the distribution of polyfunctional (E) CD4^+^ and (F) CD8^+^ T cell subsets and their dynamic shifts.
**Figure S4**: Comparison of different CD4⁺ and CD8⁺ T cell functional subsets. (A) Comparison of the polyfunctional strength index (PSI) values of effector, stimulatory, chemotactic, regulatory, and inflammatory CD4⁺ T cell subsets between the ICI‐resistant and post‐sitravatinib groups. (B) Comparison of polyfunctionality among CD4⁺ T cell subsets with different cytokine‐secreting capacities (secreting 2–5 cytokines). (C) Comparison of the PSI values of effector, stimulatory, chemotactic, regulatory, and inflammatory CD8⁺ T cell subsets between the ICI‐resistant and post‐sitravatinib groups. (D) Comparison of polyfunctionality among CD8⁺ T cell subsets with different cytokine‐secreting capacities (secreting 2–5 cytokines).
**Table S1**: Summary of adverse events that occurred during the trial.

## Data Availability

TCR‐seq data were uploaded to the Gene Expression Omnibus database (accession number GSE309446). The remaining data have been presented in this article.
