# Supplementary material for: A Phase II Study of Sitravatinib Combined With Tislelizumab Plus Docetaxel for Acquired Resistance to PD‐(L)1 in Patients With Advanced/Metastatic Non‐Small Cell Lung Cancer
Source: MedComm (2020). 2025 Dec 11;6(12):e70538. doi: 10.1002/mco2.70538 (PMC12696423; doi:10.1002/mco2.70538)
Supplement: Supplementary file 1 — Figure S1: Analysis of T cell repertoire before and after immunotherapy. (A) Comparison of clonality index and (B) Shannon diversity index in tissue samples from NSCLC patients in the treatment‐naïve (n = 5) and ICI‐resistant (n = 7) groups. (C) Bar charts showing the comparison of TRBJ and (D) TRBV gene usage frequencies between the treatment‐naïve (n = 5) and ICI‐resistant (n = 7) groups from plasma samples. (E) Bar chart displaying the distribution of CDR3aa sequence lengths between the treatment‐naïve (n = 5) and ICI‐resistant (n = 7) groups from tissue samples. Figure S2: Analysis of CDR3 amino acid sequences. (A) Bar chart showing the number of shared CDR3aa sequences derived from varying numbers of samples across different patients in the ICI‐resistant and post‐sitravatinib groups. (B) Representative motifs of shared CDR3aa sequences in the ICI‐resistant and post‐sitravatinib groups. (C) Bar chart comparing the frequencies of major shared CDR3aa motifs in paired patient samples between the ICI‐resistant and post‐Sitravatinib groups. (D) Scatter plots illustrating the distribution of clones between the ICI‐resistant and post‐Sitravatinib groups from plasma samples, presented sequentially for patients 01003, 01004, 01005, 01006, and 01007. Figure S3: Polyfunctionality analysis of CD4+ and CD8+ T cells before and after immunotherapy treatment. (A) Comparison of the polyfunctional strength index (PSI) of peripheral CD4+ T cells in the treatment‐naïve and ICI‐resistant groups. (B) Comparison of the polyfunctionality of peripheral CD4+ T cells. (C) Comparison of the PSI of peripheral CD8+ T cells in the treatment‐naïve and ICI‐resistant groups. (D) Comparison of the polyfunctionality of peripheral CD8+ T cells. PAT‐PCA plots illustrating the distribution of polyfunctional (E) CD4+ and (F) CD8+ T cell subsets and their dynamic shifts. Figure S4: Comparison of different CD4⁺ and CD8⁺ T cell functional subsets. (A) Comparison of the polyfunctional strength index (PSI) [file MCO2-6-e70538-s001.docx]

**Supplementary Materials**

**A phase** **II study of** **sitravatinib** **combined with** **tislelizumab plus** **docetaxel for acquired resistance to PD-(L)1 in patients with** **advanced/metastatic** **non-small cell lung cancer**

**Running title: A phase II study of anti-PD-(L)1-resistant NSCLC**

Yalun Li^1,2#^, Jin Zhou^3#^, Li Jiang^4#^, Hua Xie^3^, Zonglian Gong^4^, Ke Wang^1^, Yan Zhang^2^, Yan Li^2^, Weimin Li^1*^, Panwen Tian^1,2*^

^1^Department of Pulmonary and Critical Care Medicine, State Key Laboratory of Respiratory Health and Multimorbidity, Precision Medicine Key Laboratory of Sichuan Province, West China Hospital, Sichuan University, China;

^2^Lung Cancer Centre/Lung Cancer Institute, West China Hospital, Sichuan University, China;

^3^Department of Medical Oncology, Sichuan Clinical Research Centre for Cancer, Sichuan Cancer Hospital & Institute, Sichuan Cancer Centre, Affiliated Cancer Hospital of University of Electronic Science and Technology, China;

^4^Department of Pulmonary and Critical Care Medicine, Affiliated Hospital of North Sichuan Medical College, China.

^#^These authors contributed equally to this work and share first authorship.

^*^**Corresponding Authors**

Panwen Tian, E-mail: mrascend@163.com;

Weimin Li, E-mail: weimin003@163.com

West China Hospital, Sichuan University, 37 Guoxue Alley, Wuhou District, Chengdu, Sichuan Province, 610041, China.


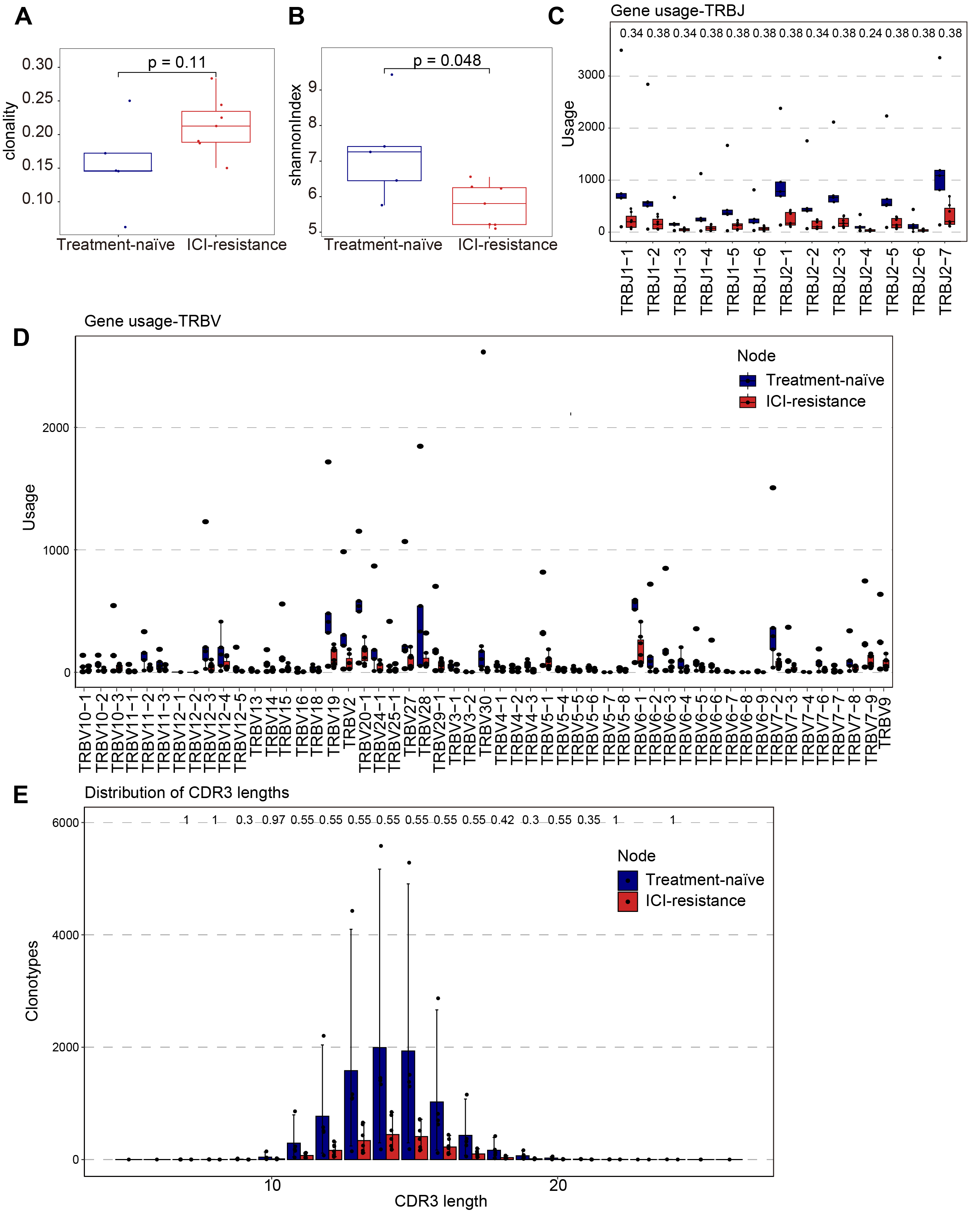


**Figure S1. Analysis of T-cell repertoire before and after immunotherapy.** (A) Comparison of clonality index and (B) Shannon diversity index in tissue samples from NSCLC patients in the treatment-naïve (n = 5) and ICI-resistance (n = 7) groups. (C) Bar charts showing the comparison of TRBJ and (D) TRBV gene usage frequencies between the treatment-naïve (n = 5) and ICI-resistance (n = 7) groups from plasma samples. (E) Bar chart displaying the distribution of CDR3aa sequence lengths between the treatment-naïve (n = 5) and ICI-resistance (n = 7) groups from tissue samples.


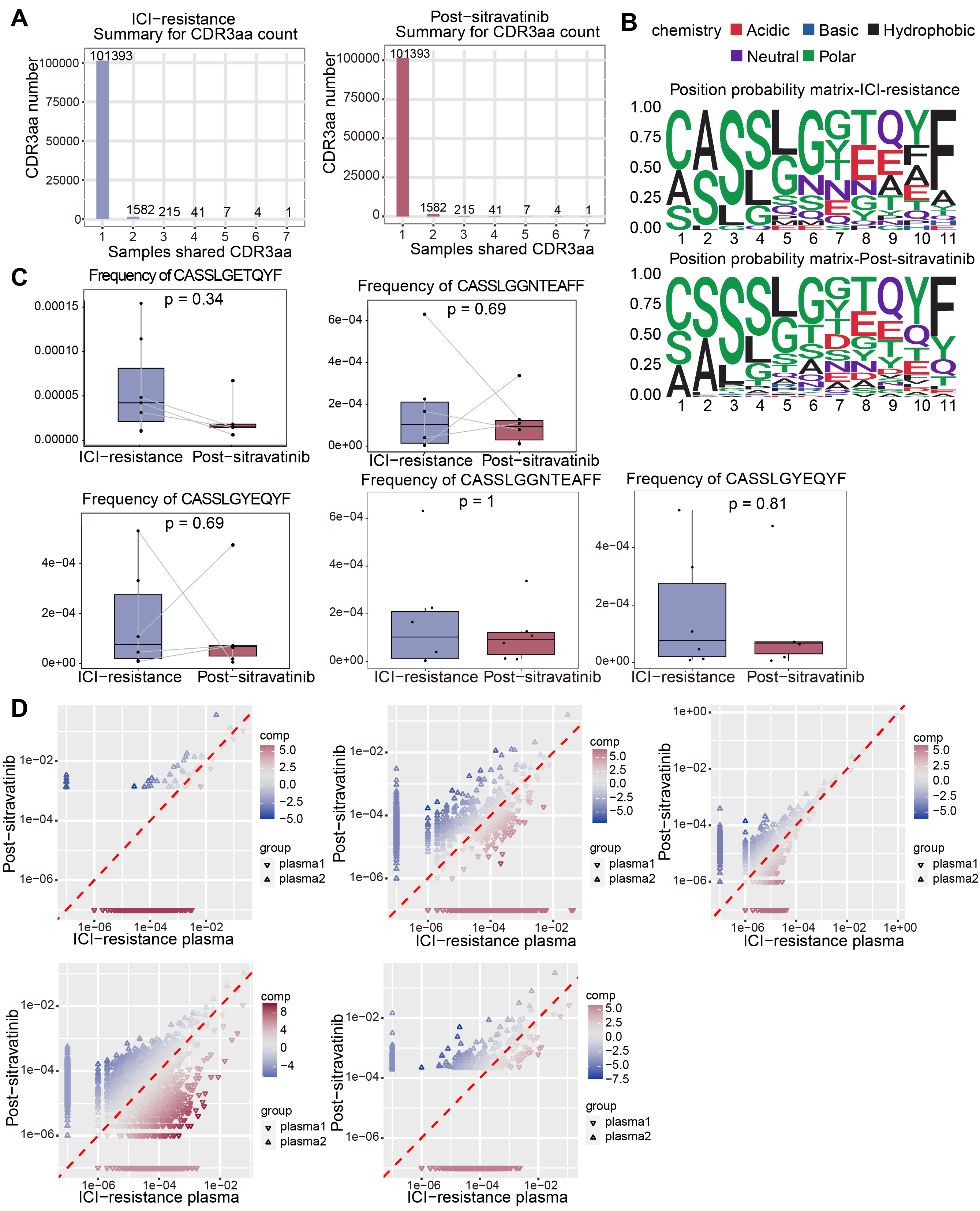


**Figure 2. Analysis of CDR3 amino acid sequences.** (A) Bar chart showing the number of shared CDR3aa sequences derived from varying numbers of samples across different patients in the ICI-resistance and post-sitravatinib groups. (B) Representative motifs of shared CDR3aa sequences in the ICI-resistance and post-sitravatinib groups. (C) Bar chart comparing the frequencies of major shared CDR3aa motifs in paired patient samples between the ICI-resistance and post-Sitravatinib groups. (D) Scatter plots illustrating the distribution of clones between the ICI-resistance and post-Sitravatinib groups from plasma samples, presented sequentially for patients 01003, 01004, 01005, 01006, and 01007.


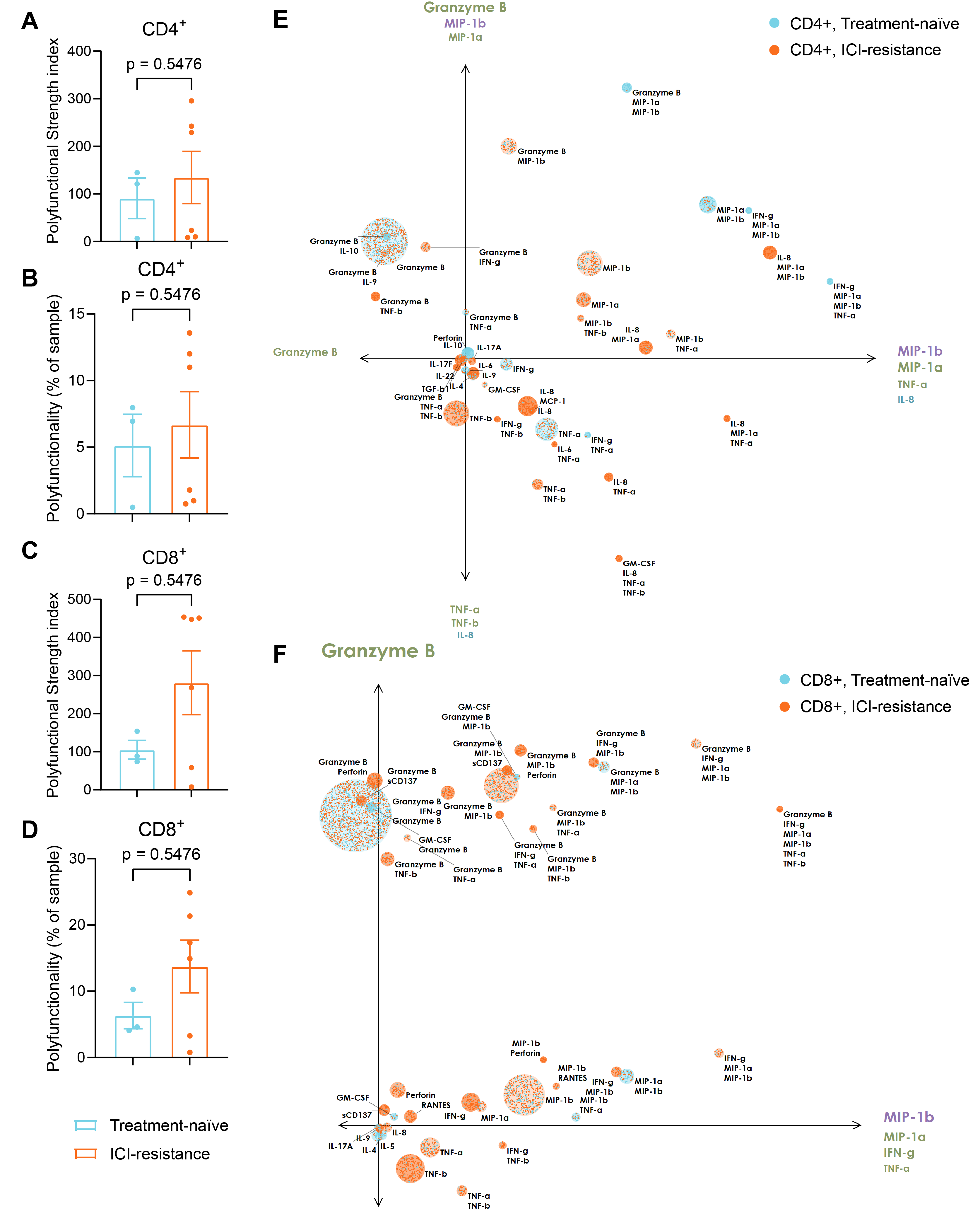


**Figure S3. Polyfunctionality analysis of CD4^+^ and CD8^+^ T cells before and after immunotherapy treatment.** (A) Comparison of the polyfunctional strength index (PSI) of peripheral CD4^+^ T cells in the treatment-naïve and ICI-resistant groups. (B) Comparison of the polyfunctionality of peripheral CD4^+^ T cells. (C) Comparison of the PSI of peripheral CD8^+^ T cells in the treatment-naïve and ICI-resistant groups. (D) Comparison of the polyfunctionality of peripheral CD8^+^ T cells. PAT-PCA plots illustrating the distribution of polyfunctional (E) CD4^+^ and (F) CD8^+^ T cell subsets and their dynamic shifts.


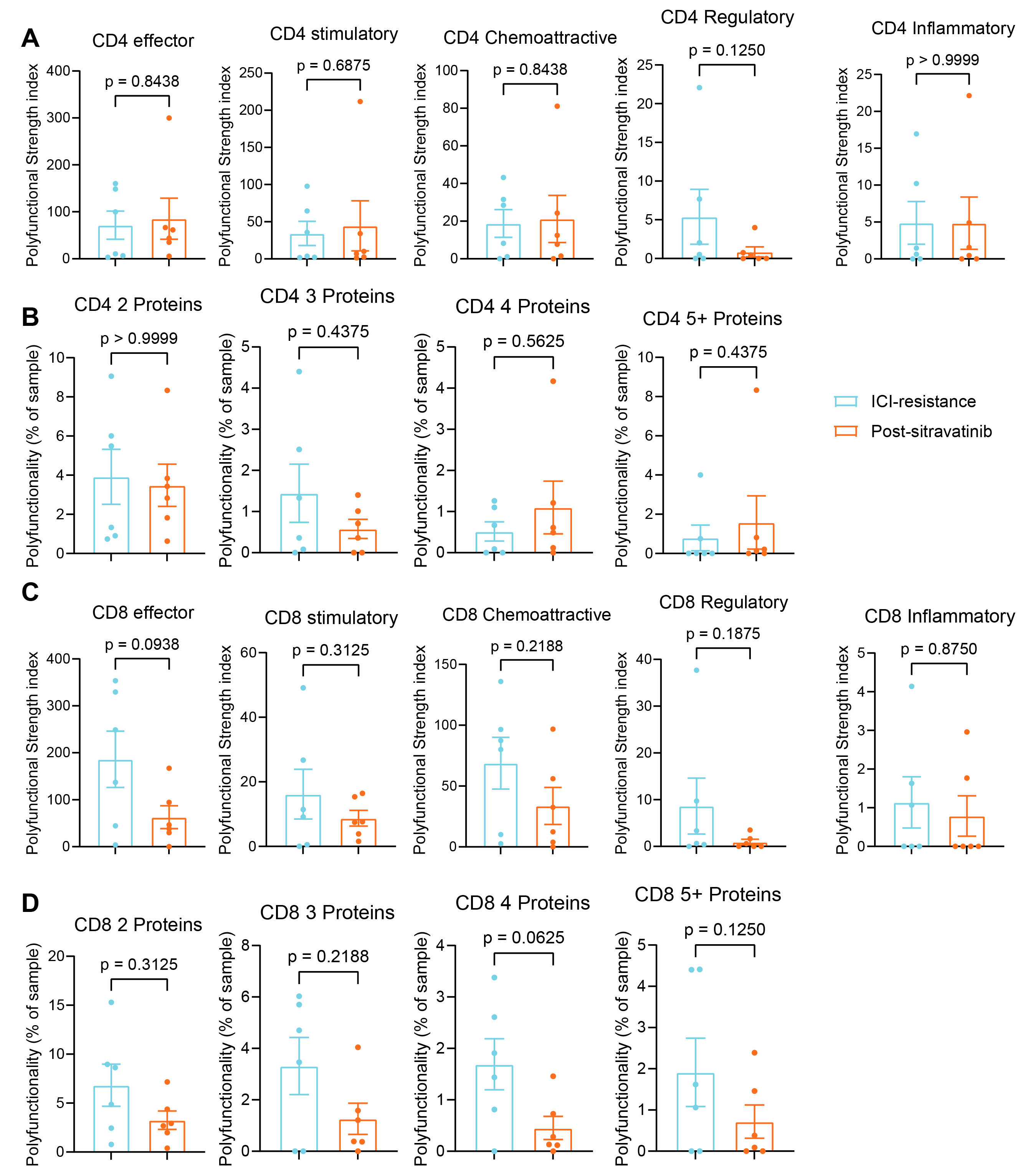


**Figure S4. Comparison of different CD4⁺ and CD8⁺ T-cell functional subsets.**

(A) Comparison of the polyfunctional strength index (PSI) values of effector, stimulatory, chemotactic, regulatory, and inflammatory CD4⁺ T cell subsets between the ICI-resistant and post-sitravatinib groups. (B) Comparison of polyfunctionality among CD4⁺ T cell subsets with different cytokine-secreting capacities (secreting 2-5 cytokines). (C) Comparison of the PSI values of effector, stimulatory, chemotactic, regulatory, and inflammatory CD8⁺ T cell subsets between the ICI-resistant and post-sitravatinib groups. (D) Comparison of polyfunctionality among CD8⁺ T cell subsets with different cytokine-secreting capacities (secreting 2-5 cytokines).

**Table S1. Summary of adverse events that occurred during the trial.**

| **Preferred TEAE** | **N (%) N = 13** | | | | | |
| --- | --- | --- | --- | --- | --- | --- |
| **TRAE** | 13(100) | | | | | |
| **≥G3 TRAE** | 12 (92.3) | | | | | |
| TEAE leading to interruption/dose modification of any study treatment | 10 (76.9) | | | | | |
| TEAE leading to permanent discontinuation of any study treatment | 1 (7.7) | | | | | |
| **SAE** | 6 (46.2) | | | | | |
| Treatment–related SAE | 2 (15.4) | | | | | |
| **irAE** | 4 (30.8) | | | | | |
| **TRAE reported in ≥2 patients** | **G1** | **G2** | **G3** | **G4** | **G5** | **Any Grade** |
| Any TRAE | 13(100) | 11(84.6) | 10(76.9) | 7(53.8) | 2(15.4) | 13(100) |
| Neutrophil count decreased | 0 | 3(23.1) | 3(23.1) | 6(46.2) | 0 | 12(92.3) |
| WBC count decreased | 2(15.4) | 4(30.8) | 4(30.8) | 1(7.7) | 0 | 11(84.6) |
| ALT increased | 9(69.2) | 0 | 1(7.7) | 0 | 0 | 10(76.9) |
| AST increased | 10(76.9) | 0 | 0 | 0 | 0 | 10(76.9) |
| Rash | 9(69.2) | 0 | 0 | 0 | 0 | 9(69.2) |
| Alopecia | 8(61.5) | 1(7.7) | 0 | 0 | 0 | 9(69.2) |
| Diarrhoea | 6(46.2) | 0 | 2(15.4) | 0 | 0 | 8(61.5) |
| Dysphonia | 6(46.2) | 2(15.4) | 0 | 0 | 0 | 8(61.5) |
| Fatigue | 6(46.2) | 0 | 0 | 0 | 0 | 6(46.2) |
| Anaemia | 4(30.8) | 2(15.4) | 0 | 0 | 0 | 6(46.2) |
| Hand-foot syndrome | 2(15.4) | 1(7.7) | 3(23.1) | 0 | 0 | 6(46.2) |
| Constipation | 5(38.5) | 0 | 0 | 0 | 0 | 5(38.5) |
| Stomatitis | 3(23.1) | 2(15.4) | 0 | 0 | 0 | 5(38.5) |
| Proteinuria | 1(7.7) | 2(15.4) | 1(7.7) | 0 | 0 | 4(30.8) |
| GGT increased | 3(23.1) | 1(7.7) | 0 | 0 | 0 | 4(30.8) |
| CK-MB increased | 4(30.8) | 0 | 0 | 0 | 0 | 4(30.8) |
| Dry mouth | 4(30.8) | 0 | 0 | 0 | 0 | 4(30.8) |
| Lymphocyte count decreased | 4(30.8) | 0 | 0 | 0 | 0 | 4(30.8) |
| Epistaxis | 3(23.1) | 0 | 0 | 0 | 0 | 3(23.1) |
| TSH increased | 3(23.1) | 0 | 0 | 0 | 0 | 3(23.1) |
| Hypoalbuminaemia | 2(15.4) | 1(7.7) | 0 | 0 | 0 | 3(23.1) |
| Nausea | 3(23.1) | 0 | 0 | 0 | 0 | 3(23.1) |
| Hypophosphataemia | 2(15.4) | 0 | 0 | 0 | 0 | 2(15.4) |
| Abdominal pain | 1(7.7) | 1(7.7) | 0 | 0 | 0 | 2(15.4) |
| Abdominal distension | 2(15.4) | 0 | 0 | 0 | 0 | 2(15.4) |
| Hypercholesterolaemia | 2(15.4) | 0 | 0 | 0 | 0 | 2(15.4) |
| Hypertriglyceridaemia | 2(15.4) | 0 | 0 | 0 | 0 | 2(15.4) |
| Hypothyroidism | 0 | 2(15.4) | 0 | 0 | 0 | 2(15.4) |
| Immune-mediated pneumonitis | 0 | 0 | 1(7.7) | 0 | 1(7.7) | 2(15.4) |
| LDH increased | 2(15.4) | 0 | 0 | 0 | 0 | 2(15.4) |
| Lower-limb pain | 2(15.4) | 0 | 0 | 0 | 0 | 2(15.4) |
| Haemoptysis | 2(15.4) | 0 | 0 | 0 | 0 | 2(15.4) |
| Dizziness | 2(15.4) | 0 | 0 | 0 | 0 | 2(15.4) |
| Blood pressure increased | 1(7.7) | 0 | 1(7.7) | 0 | 0 | 2(15.4) |
| Low-back pain | 2(15.4) | 0 | 0 | 0 | 0 | 2(15.4) |
| Pneumonia | 0 | 0 | 0 | 1(7.7) | 1(7.7) | 2(15.4) |

TEAE, treatment-emergent adverse event; TRAE, treatment-related adverse event, SAE: serious adverse event; irAE, immune-related adverse event; WBC, white blood cell; ALT, alanine aminotransferase; AST, aspartate aminotransferase; GGT, gamma-glutamyltransferase; CK-MB, creatine kinase MB; TSH, thyroid-stimulating hormone; LDH, lactate dehydrogenase.
